# Investigating Immune Responses to the scAAV9-*HEXM* Gene Therapy Treatment in Tay–Sachs Disease and Sandhoff Disease Mouse Models

**DOI:** 10.3390/ijms22136751

**Published:** 2021-06-23

**Authors:** Shalini Kot, Subha Karumuthil-Melethil, Evan Woodley, Violeta Zaric, Patrick Thompson, Zhilin Chen, Erik Lykken, John G. Keimel, William F. Kaemmerer, Steven J. Gray, Jagdeep S. Walia

**Affiliations:** 1 Department of Biomedical and Molecular Sciences, Queen’s University, Kingston, ON K7L 3N6, Canada; 11sk82@queensu.ca (S.K.); evan.woodley@gmail.com (E.W.); 2 Gene Therapy Center, University of North Carolina at Chapel Hill, Chapel Hill, NC 27599, USA; subha.km@gmail.com (S.K.-M.); violetav@email.unc.edu (V.Z.); erik_lykken@med.unc.edu (E.L.); Steven.Gray@UTSouthwestern.edu (S.J.G.); 3 Department of Pediatrics, University of Texas Southwestern Medical Center, Dallas, TX 75390, USA; 4 Medical Genetics, Department of Pediatrics, Queen’s University, Kingston, ON K7L 2V7, Canada; pt24@queensu.ca (P.T.); zc@queensu.ca (Z.C.); 5 New Hope Research Foundation, North Oaks, MN 55127, USA; jack.keimel@newhoperesearch.org (J.G.K.); bill.kaemmerer@newhoperesearch.org (W.F.K.)

**Keywords:** GM2, gangliosidosis, Tay-Sachs, Sandhoff, scAAV9-*HEXM*, HexM, immunocompetence, transgene, murine, capsid

## Abstract

GM2 gangliosidosis disorders are a group of neurodegenerative diseases that result from a functional deficiency of the enzyme β-hexosaminidase A (HexA). HexA consists of an α- and β-subunit; a deficiency in either subunit results in Tay–Sachs Disease (TSD) or Sandhoff Disease (SD), respectively. Viral vector gene transfer is viewed as a potential method of treating these diseases. A recently constructed isoenzyme to HexA, called HexM, has the ability to effectively catabolize GM2 gangliosides in vivo. Previous gene transfer studies have revealed that the scAAV9-*HEXM* treatment can improve survival in the murine SD model. However, it is speculated that this treatment could elicit an immune response to the carrier capsid and “non-self”-expressed transgene. This study was designed to assess the immunocompetence of TSD and SD mice, and test the immune response to the scAAV9-*HEXM* gene transfer. HexM vector-treated mice developed a significant anti-HexM T cell response and antibody response. This study confirms that TSD and SD mouse models are immunocompetent, and that gene transfer expression can create an immune response in these mice. These mouse models could be utilized for investigating methods of mitigating immune responses to gene transfer-expressed “non-self” proteins, and potentially improve treatment efficacy.

## 1. Introduction

GM2 gangliosidoses are a group of inherited lysosomal storage diseases, characterized by the toxic accumulation of GM2 gangliosides in the brain. This is the result of a deficiency of the β-hexosaminidase A (HexA) enzyme involved in the catabolism of these lipids [1]. HexA is a heterodimer consisting of an α- and β-subunit [2]. Mutations in either of these genes can produce a HexA deficiency. Tay–Sachs Disease (TSD) results from a deficient α-subunit [3]; a deficient β-subunit leads to Sandhoff Disease (SD) [1]. Both TSD and SD have very similar clinical phenotypes. Between three to five months of age, patients with the infantile-onset form of these diseases typically show signs of impaired motor function, decreased attentiveness, visual impairment, and rapid mental deterioration [4]. Patients with the infantile-onset form commonly die before four years of age. Juvenile-onset and adult forms of the disease are less severe, with a slower deterioration [5]. There is currently no successful treatment for GM2 gangliosidoses.

The murine TSD model was generated by knockout of the mouse *Hexa* gene, encoding for the α-subunit of HexA, thereby resulting in deficient HexA activity. TSD mice show GM2 build-up in certain regions of the brain; however, they do not demonstrate a significant neurological decline. This could be due an alternative metabolic pathway in mice, the sialidase pathway, which can catabolize GM2 gangliosides to GA2 [6]. These lipids can then be broken down to glucosylceramide by HexB (ββ homodimer). As TSD mice still retain their HexB activity, they can maintain the GM2 lipid accumulation below toxic levels, without the need for HexA. The SD mouse model was created by knock-out of the *Hexb* gene, encoding for the β-subunit. SD mice have a deficiency in both HexA and HexB enzyme activity. In contrast to the TSD mouse model, SD mice display extensive GM2 accumulation in the brain, showing symptoms similar to the infantile-onset form of the disease, with impaired behavioral function, muscle weakness, tremors, and ataxia [7]. Multiple studies have been performed using each of these mouse models to further comprehend the mechanisms of the diseases, and to find strategies to treat them.

The immunocompetence of TSD and SD mice has been questioned in previous literature. The accumulation of lipids in glycosphingolipid (GSL) lysosomal storage disorders, including GM2 gangliosidoses, has been shown to impair the selection and function of invariant natural killer T cells (*i*NKT cells), a subset of T lymphocytes that help mediate regulatory immune function [8]. Immunological alterations in the thymus of SD mice have also been observed in the severe progressive stage of the neurological disease, past 15 weeks of age [9]. The development of autoimmune responses is evident in these mice, with a decrease in immature CD4^+^/CD8^+^ T cells and an increase in CD4^+^/CD8^−^ T cells in the thymus, as well as the upregulation of genes associated with immune responses. Since it was not clearly proven before that TSD and SD mice can mount an immune response to a foreign protein, it was decided that the immunocompetence of these mouse models must be assessed. This was evaluated in this study by investigating the B and T cell immune responses of both TSD and SD mice to the expression of a human transgene, *HEXM*.

There is a history of utilizing adeno-associated viral vectors (AAV) to treat TSD and SD in preclinical models [10,11,12,13]. Important for central nervous system (CNS) gene therapy applications, AAV can transduce non-dividing cells and can confer long-term stable gene expression without associated inflammation or toxicity [14,15,16,17]. Several groups have shown that AAV serotype 9 (AAV9) vectors can achieve broad distribution across the CNS, and provide dramatic therapeutic benefit to neurological disorders using intravenous (IV) and cerebrospinal (CSF) routes of delivery [18,19,20,21,22,23,24]. Therapeutic approaches utilizing AAV9 with IV or CSF administration may scale and translate more effectively from mice to larger animal models, than direct parenchymal vector delivery [18,22,25,26,27,28]. Many clinical trials using AAV9 in the treatment of varying CNS disorders have been initiated (Giant Axonal Neuropathy, intrathecal [IT] administration, clinicaltrials.gov NCT02362438); MPS IIIA (IV administration, clinicaltrials.gov NCT02716246); Neuronal Ceroid Lipofuscinosis Type 6 (IT administration, clinicaltrials.gov NCT02725580)), such that AAV9 now has a track record of human application. The AAV9-mediated therapy for the treatment of Spinal Muscular Atrophy (clinicaltrials.gov NCT02122952) has been approved by the U.S. Food and Drug Administration (Zolgensma^®^ FDA [29]). The immune response to the AAV9 capsid in TSD mice was also assessed in this study.

A recently constructed isoenzyme to the human HexA heterodimer, called HexM, has the ability to efficiently catabolize GM2 gangliosides in vivo [30]. The HexM enzyme consists of two μ-subunits. This μ-subunit was engineered using the 529 amino acid sequence of the human β-hexosaminidase α-subunit as a base, with 21 amino-acid substitutions and one deletion made to incorporate the stable dimer interface and GM2AP binding interface of the human β-hexosaminidase β-subunit [30]. The HexM coding gene, *HEXM*, has a size of 1.6 kb, and thus can be packaged in a self-complementary AAV viral genome configuration, with short promoter and polyA regulatory sequences. Self-complementary AAV vectors can afford >10-fold higher gene transfer efficiency, versus traditional single-stranded AAV vectors [25,31,32]. Previous pre-clinical gene transfer studies have shown that the scAAV9-*HEXM* treatment reduces GM2 ganglioside levels in the brain, and improves survival in mouse models [12,23].

Although the field of gene therapy has progressed immensely over the years, one of the challenges faced is the potential for patients to develop an immunological response to the treatment [33,34]. Gene therapy treatments have been found to trigger undesirable T and B cell immune responses in previous studies [35,36,37,38,39], especially when the therapeutic protein or viral vector is seen as foreign by the host immune system. These immune responses could contribute to a failure of the gene therapy treatment to provide long-term benefits. Therefore, when studying treatment options for genetic disorders using gene therapy, it is crucial to have an animal model that could allow one to investigate the impact of an immune response to the transgene or viral capsid.

As the HexM protein is a hybrid variant of the human β-hexosaminidase α- and β-subunits, it has the potential to create novel “non-self” immune responses. In the present study, we confirmed the immunocompetency of TSD and SD mice, and tested the immune response towards intravenous delivery of scAAV9-*HEXM.* Sera were analyzed for antibodies to the vector or transgene product, and lymph node cells and splenocytes were analyzed for the production of the cytokine interferon-γ (IFN-γ). Analysis of the vector biodistribution of the *HEXM* gene was performed to assess the stability of transduced cells in the brain and non-CNS tissues. It was found that the scAAV9-*HEXM* treatment induced a T and B cell immune response to the viral capsid and the expressed human protein in TSD and SD mice.

## 2. Results

To evaluate the immunocompetency of SD and TSD mouse models, and the immune response to gene therapy treatment, adult heterozygous and homozygous knockout mice were injected intravenously with either scAAV9-*HEXM*, HexM purified protein (+/−adjuvant), or vehicle (+/−adjuvant). Experimental mice were given either one injection or two, 3 weeks apart. The second injection may have varied from the first injection, depending on the treatment group. TSD mice were euthanized 3 or 6 weeks post injection 1. SD mice were euthanized either 3 weeks or 9 weeks post injection 1. For a detailed description of the study design, refer to the Study Design section (Section 4.3) of the Materials and Methods (Section 4).

### 2.1. Cellular Immune Response to scAAV9-HEXM Gene Therapy

#### 2.1.1. HexM-Specific T Cell Response in Splenocytes of Tay Sachs Disease (TSD) Mice

**Figure 1:**
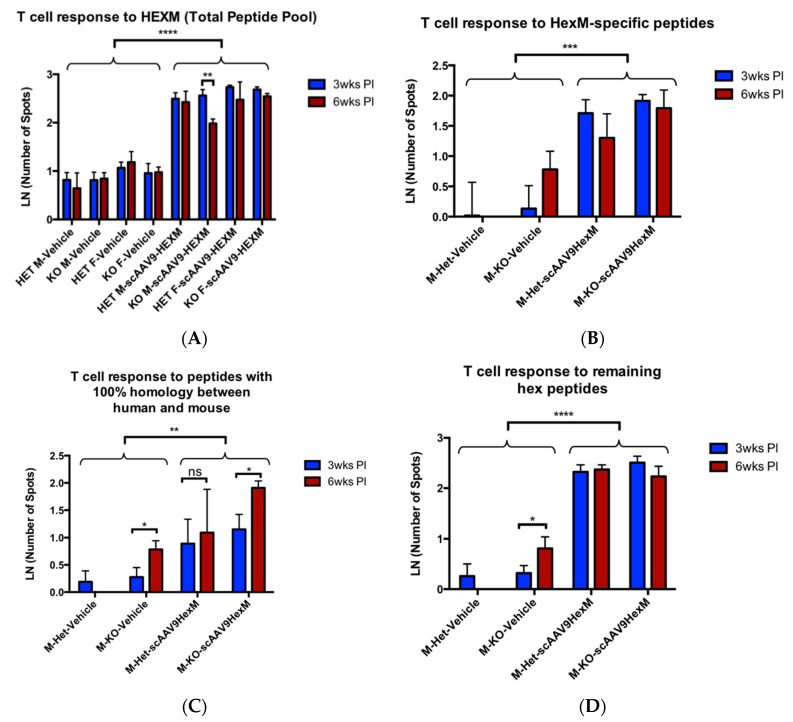
Hex peptide T cell responses in TSD mice. (**A**) ELISpot IFN-γ secretion in response to the total HexM 52 peptide pool (See Supplementary Materials) in Het and KO TSD mice 3 weeks and 6 weeks post injection (PI). HEXM-treated mice showed significantly higher number of spots to HexM protein compared to vehicle-treated mice (ANOVA main effect of treatment, *p* < 0.0001). (**B**) Comparison of IFN-γ secretion spots in response to novel HexM peptides (i.e., not homologous with either the mouse or human HexA) in male Het and KO TSD mice 3 weeks and 6 weeks. *HEXM*-treated mice showed significantly higher number of spots to HexM protein compared to vehicle-treated mice (ANOVA main effect of treatment, *p* < 0.001). (**C**) Number of spots in response to exposure with peptides with 100% homology between humans and mice in male Het and KO TSD mice 3 weeks and 6 weeks PI. *HEXM*-treated mice showed significantly higher number of spots to HexM protein compared to vehicle-treated mice (ANOVA main effect of treatment, *p* < 0.01), and there was a tendency for KO TSD mice to show a greater response to these peptides at 6 weeks than at 3 weeks (*p* < 0.05, post hoc tests). (**D**) Number of spots in response to the HexM peptide pool specific to human HexA in male Het and KO TSD mice 3 weeks and 6 weeks PI. *HEXM*-treated mice showed significantly higher number of spots to HexM protein compared to vehicle-treated mice (ANOVA main effect of treatment, *p* < 0.001). In summary, both heterozygous and KO TSD mice treated with scAAV9-*HexM* had a T-cell response to all types of peptides in the hex peptide pool. M = male, F = female, wks = weeks, PI = post injection, N per group = 3, error bars = SEM, **** = *p* < 0.0001, *** = *p* < 0.001, ** = *p* < 0.01, * = *p* < 0.05.

Interferon-γ Enzyme-Linked Immunospot (ELISpot) assays were carried using splenocytes and pooled lymphocytes of treated experimental mice. The cells were treated with HexM peptides, which were used as a stimulant to assess the HexM-specific T cell response to the gene therapy treatment. Figure 1A reveals that the delivery of scAAV9-*HEXM* did produce T cells that responded to the HexM peptide library described in the Materials and Methods. This was the case in both male and female TSD Het and KO mice, with significantly more T-cells secreting IFN-γ cytokine when stimulated by the total HexM peptide pool among splenocytes from *HEXM*-treated mice, compared to control mice given vehicle injections (*p* < 0.0001). The assay was repeated using cryo-preserved splenocytes from male mice from both time points (*n* = 24), to compare the T cell response to the various pools of HexA peptides. The first pool contained 15 HexM-specific peptides, the second pool consisted of five mouse–human 100% homology peptides, and the third pool consisted of the remaining 32 peptides specific to human HexA (for a full description, refer to the Materials and Methods (Section 4)). Figure 1 panels B–D reveal the data for this experiment. In both TSD Het and KO mice injected with scAAV9-*HEXM*, an anti-peptide T cell response occurred across HexM-specific peptides (Figure 1B), peptides with 100% homology between mice and humans (Figure 1C), and all remaining Hex peptides as well (Figure 1D). The results indicate that the scAAV9-*HEXM* delivery induced a T cell response to HexM, as well as to the non-HexM specific peptides in both Het and KO TSD mice, thus confirming the intact immune response in TSD mice.

#### 2.1.2. HexM-Specific T Cell Response in Splenocytes of Sandhoff Disease (SD) Mice

**Figure 2:**
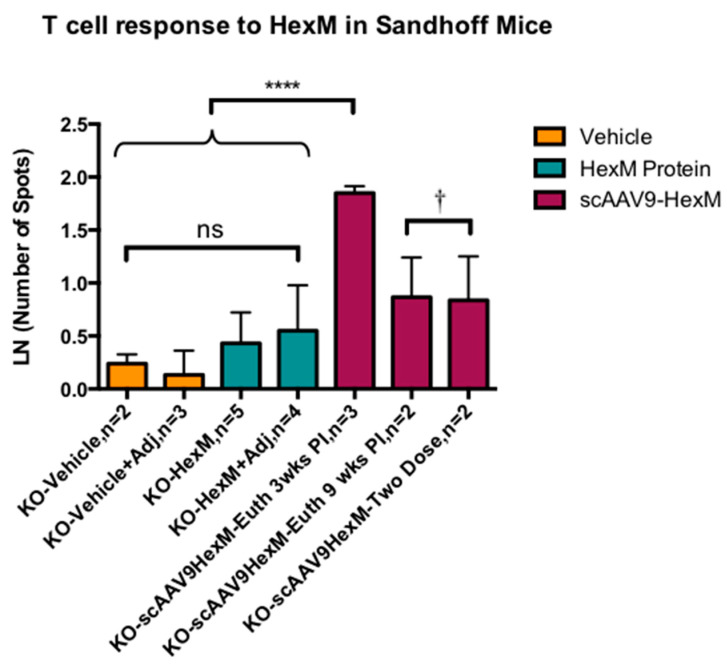
HexM-specific T cell response in SD mice. ELISPot IFN-γ secretion in response to HexM stimulated splenocytes of SD mice 3 weeks and 9 weeks post injection. Mice receiving scAAV9-HexM and euthanized 3 weeks post injection showed significantly higher number of spots in response to HexM peptides, compared to SD mice receiving vehicle or HexM protein injections only, with or without adjuvant (*p* < 0.0001); differences among the latter groups of SD mice were not significant. †Mice receiving one or two injections of scAAV9-HexM and euthanized 9 weeks post the first (or only) injection, had fewer spots than mice receiving scAAV9-HexM euthanized 3 weeks post injection (*p* < 0.01 post hoc), but a higher number of spots than mice receiving vehicle or vehicle with adjuvant (*p* = 0.06 post hoc using Tukey–Kramer corrections for p values.) KO = knock out SD mice, Adj = adjuvant, Euth = euthanized, wks = weeks, PI = post injection, error bars = SEM, n per group as shown, ns = not significant, **** = *p* < 0001.

The ELISpot assay was also performed using splenocytes obtained from SD experimental mice. These cells were treated with the complete HexM peptide library (52 peptides total). The splenocyte analysis in the SD mice (Figure 2) also revealed a significantly higher HexM specific IFN-γ response, in terms of number of spots, in the KO mice treated with scAAV9-*HEXM* at 6 weeks and then euthanized three weeks post injection, compared to mice injected with vehicle or HexM protein, with or without adjuvant (Figure 2). The mice treated with vehicle or purified HexM protein, with or without adjuvant, showed low numbers of splenocytes producing IFN-γ upon stimulation by the complete HexM 52 peptide pool. Mice treated with scAAV9-*HEXM* and then euthanized 9 weeks post injection, or mice treated with the vector treatment twice and then euthanized 6 weeks post injection 2 showed low numbers of splenocytes responding to HexM at the endpoint, but higher numbers than mice injected with vehicle. These results indicate that the scAAV9-*HEXM* treatment does prompt a T cell response to the HexM peptides in SD mice, and that it is more pronounced at three weeks post injection compared to nine weeks post injection.

#### 2.1.3. AAV9 Capsid-Specific T Cell Response in TSD Mice

**Figure 3:**
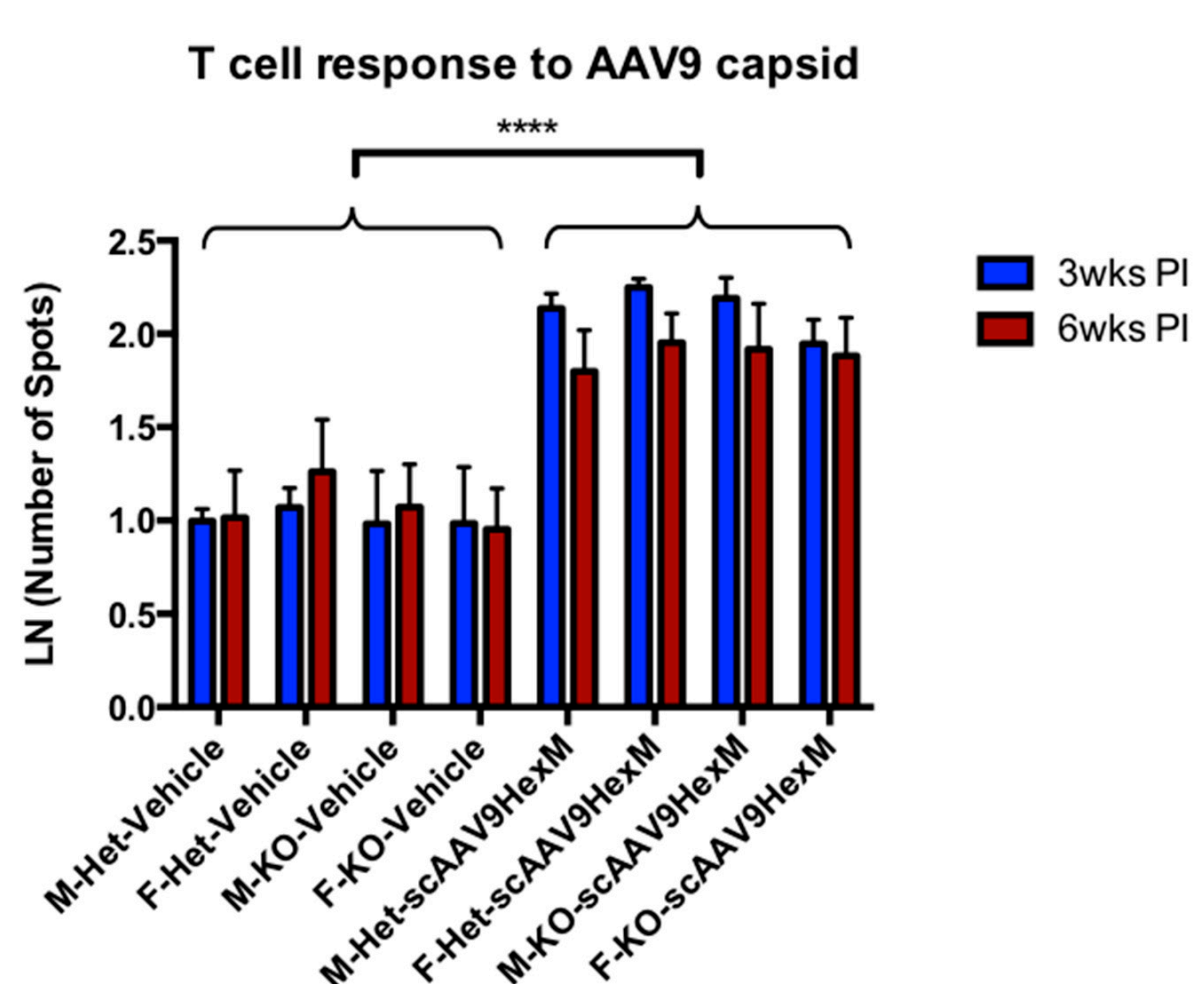
ELISpot IFN-γ secretion in response to AAV9 capsid peptides in male and female Het and KO TSD mice 3 weeks and 6 weeks post vehicle or scAAV9-*HEXM* injection (PI). Mice receiving scAAV9-*HEXM* showed significantly higher number of spots to AAV9 capsid peptides compared to vehicle-injected mice (ANOVA main effect of treatment, *p* < 0.0001). Differences between males versus females and Het versus KO mice were not statistically significant. A post hoc analysis found that among scAAV9-*HEXM*-treated mice, the T cell response in splenocytes harvested from mice at 6 weeks was less than that in splenocytes harvested from mice at 3 weeks (blue versus red bars, right half of graph, *p* < 0.01). M = male, F = female, wks = weeks, PI = post injection. Error bars = SEM, N per group = 3, **** = *p* < 0001.

The ELISpot method was repeated using an AAV9 capsid peptide library as a stimulant, to evaluate the presence of a T cell-specific immune response to the viral capsid. The T cell response to the AAV9 capsid protein in TSD mice, after delivery of scAAV9-*HEXM*, is shown in Figure 3. In both male and female Het and KO mice, IFN-γ secretion was significantly higher in scAAV9-*HEXM*-treated mice, compared to delivery of vehicle at both time points.

### 2.2. Humoral Immune Response to scAAV9-HEXM Gene Therapy

#### 2.2.1. Presence of Anti-HexM Antibodies in Sera of TSD Mice

**Figure 4:**
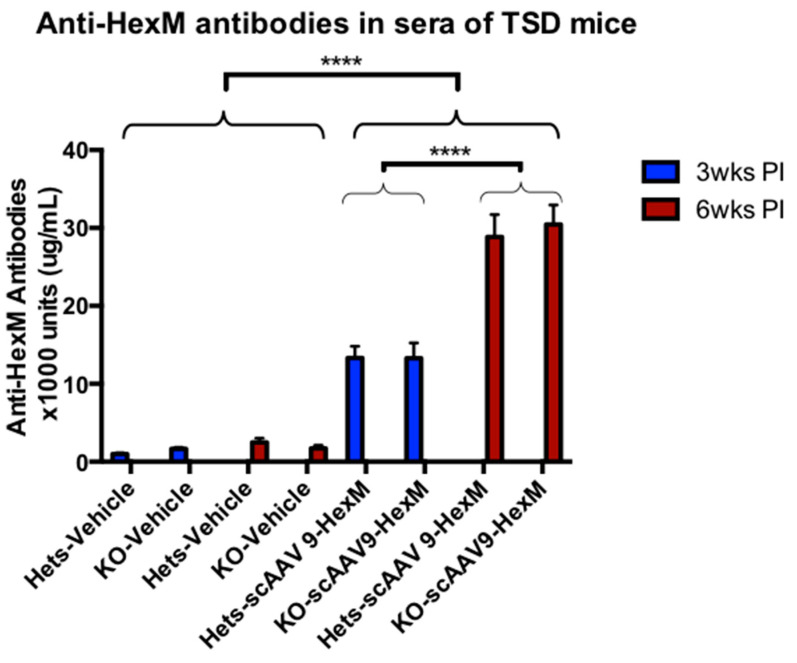
Anti-HexM antibody levels in the sera of TSD mice. HexM-specific B cell response in TSD KO and Het mice at 3 weeks and 6 weeks post scAAV9-*HEXM* injections. TSD mice given vehicle injections did not develop anti-HexM antibodies in the sera. Mice treated with scAAV9-*HEXM* developed significantly higher levels of anti-HexM antibodies, compared to those receiving vehicle. Among mice receiving scAAV9-HexM, those receiving a second injection with HexM protein after 3 weeks, then terminated at 6 weeks post- vector injection, had antibody levels more than double those that were terminated at 3 weeks post-vector injection (*p* < 0.001). There were no significant differences between Het and KO mice, nor between males and females (not shown). wks = weeks, PI = post-vector injection, **** = *p* < 0.001, n per group = 6 (3 males and 3 females), error bars = SEM.

To investigate a potential B cell response against the expressed HexM protein, an indirect enzyme-linked immunosorbent assay (ELISA) was performed to quantify the level of anti-HexM antibodies in the sera of mice. Serum was collected for this analysis at the euthanization timepoints. For the TSD mice, this was either 3 or 6 weeks post injection 1. As shown in Figure 4, TSD mice treated with vehicle generated ‘low’ to ‘nil’ levels of anti-HexM antibodies. Compared to mice treated with vehicle, mice treated with scAAV9-*HEXM* developed a significantly higher anti-HexM antibody response. Vector-treated mice receiving a later injection of HexM protein had levels of these antibodies that were more than double the levels in mice treated with scAAV9-HexM, but not receiving an injection of HexM protein. There was no significant difference in antibody levels between TSD Hets and KO mice, indicating that the HexM epitopes elicited an antibody response, regardless of the presence or absence of endogenous mouse HexA expression.

#### 2.2.2. Presence of Anti-HexM Antibodies in Sera of SD Mice

**Figure 5:**
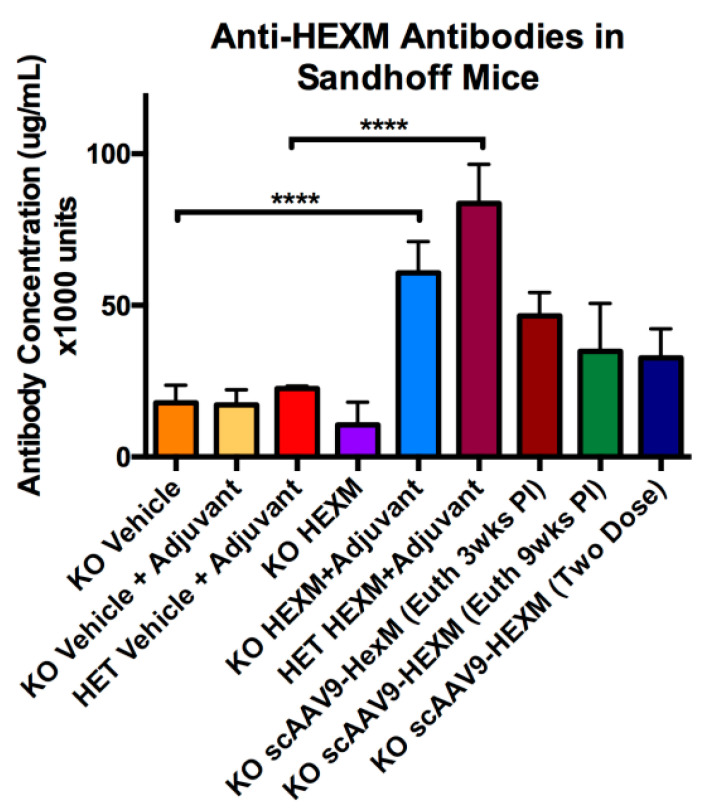
Anti-HexM antibody levels in sera of SD mice. HexM-specific B cell response in SD KO and Het mice at the endpoint (3 weeks post injection 1 or 6 weeks post injection 2). High levels of anti-HexM antibodies were observed in both Het and KO mice given HexM purified protein with adjuvant. Vehicle-injected mice showed low levels of anti-HexM antibodies. Euth = euthanized, wks = weeks, PI = post injection, error bars = SEM, **** = *p* < 0001.

The ELISA method was repeated using sera obtained from SD experimental mice. Serum was collected at the euthanization timepoint, either 3 or 9 weeks post injection 1. Figure 5 reveals the anti-HexM antibody levels in the sera of SD mice. Mice given vehicle injections, with or without adjuvant, showed low levels of anti-HexM antibodies. Significantly high levels of anti-HexM antibodies were present in both Het and KO mice injected with HexM and adjuvant, compared to mice given vehicle injections. Mice treated with scAAV9-*HEXM* showed levels of anti-HexM antibody that were intermediate, between the mice receiving HexM protein and mice receiving vehicle. Even though the HexM protein dose cannot be compared to the transgene expression level, scAAV9-*HEXM* did help stimulate an immune reaction, however less so as compared to Freund’s adjuvant.

#### 2.2.3. Neutralizing Antibodies against AAV9 Capsid in the Sera of Both TSD and SD Mice

The titer of AAV9 capsid neutralizing antibodies in the sera was determined using a cell-based assay. The expression of GFP was evaluated in Lec2 cells treated with scAAV9-CBh-GFP, that had been incubated with sera of experimental mice from this study. The results indicated that all mice injected with AAV9 vectors developed neutralizing antibodies against AAV9 (Supplementary Figure S2, Tables S2 and S3).

### 2.3. Biodistribution of scAAV9-HEXM Vector

Vector Biodistribution in the Liver and Brain in TSD Mice

**Figure 6:**
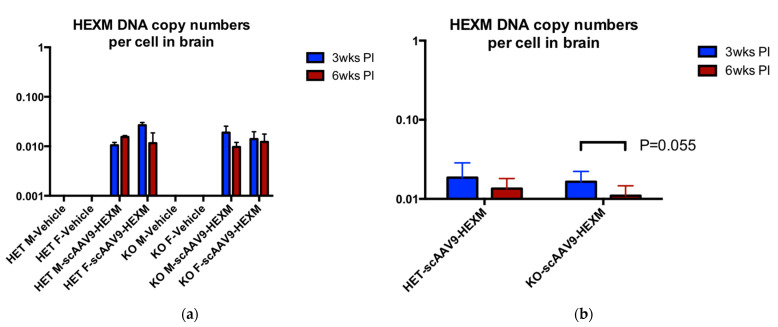
Vector biodistribution of scAAV9-*HEXM* in the brain of TSD mice. Biodistribution of the scAAV9-*HEXM* viral vector, in the brain, 3 weeks and 6 weeks post scAAV9-*HEXM* injection in TSD mice. (**a**) Comparison of scAAV9-*HEXM* treated to vehicle injected mice. (**b**) comparison of Het and KO mice. A lower biodistribution was obtained in brain samples from KO mice terminated at the later time point compared to the earlier time point, with this observation near to traditional significance levels (*p* = 0.055). M = male, F = female, wks = weeks, PI = post injection.

The biodistribution of scAAV9-*HEXM* was determined using quantitative PCR (qPCR) in liver and brain samples of TSD mice from the study. As expected, the transgene delivered by AAV9 was found in the brains of both Het and KO mice treated with scAAV9-*HEXM* (Figure 6).

**Figure 7:**
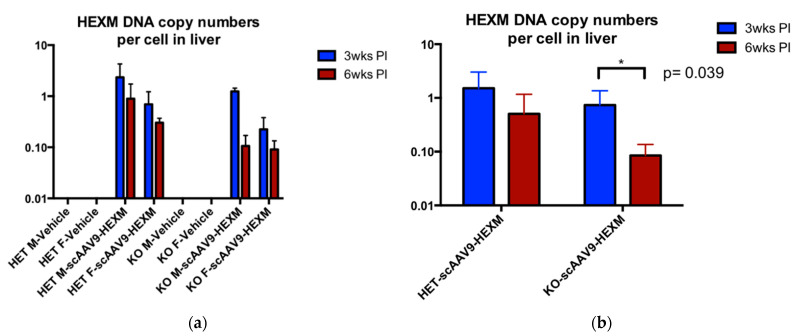
Vector biodistribution of scAAV9-*HEXM* in the liver of TSD mice. Biodistribution of the scAAV9-*HEXM* viral vector, in the liver, 3 weeks and 6 weeks post scAAV9-*HEXM* injection in TSD mice. (**a**) Comparison of scAAV9-*HEXM* treated to vehicle injected mice. (**b**) comparison of Het and KO mice. A lower biodistribution was evident in liver samples from mice terminated at the later time point compared to the earlier time point, significantly so in the KO mice (* = *p* < 0.05). M = male, F = female, wks = weeks, PI = post injection.

In the liver, across all different treatment combinations, there was a lower copy number in the liver samples from mice terminated at 6 weeks post-injection, when compared with mice terminated at 3 weeks post-injection (Figure 7). The difference was significant in the TSD KO mice that received scAAV9-*HEXM* (*p* = 0.039). These results suggest the possibility of a cytotoxic lymphocyte response to HexM leading to clearance of transduced cells in the liver. A similar biodistribution assay was done for SD mice, but no reduction was noted at 9 versus 3 weeks post-injection (data not shown).

## 3. Discussion

This study demonstrated that the scAAV9-*HEXM* gene therapy treatment initiated a T and B cell immune response to the expressed human-derived HexM enzyme, in both TSD and SD mouse models. The delivery of scAAV9-*HEXM* induced T cells that responded to the HexM PEPscreen^®^ library, and produced high levels of anti-HexM antibodies in the sera of the TSD mice, and elevated levels in the sera of SD mice. A high antibody response to purified HexM protein was observed in Het and KO SD mice in this study, when given in combination with adjuvant (Figure 5). Protein-specific immune responses to therapeutic transgenes have also been observed in other species; the induction of an immune response to a non-self-protein has been shown in rat models [40], and in non-human primates [41,42] following AAV9-mediated expression. This undesirable immune response may have a negative impact on the efficiency of the gene therapy treatment, especially in patients with null expression of the expressed protein.

The presence of T and B cell responses to HexM, in TSD and SD mice, confirms the immunocompetence of these mouse models. We did not investigate thymic alterations or natural killer (NK)-cell activity, however, as previously described for these mice [9].

In this study, some cohorts of mice had a follow-up injection of HexM-His_6_ protein with adjuvant, and a later termination time point, in order to investigate a stimulated response. It was hypothesized that as a result of this challenge, the response in these cohorts would be stronger. This was indeed the case for the antibody response in TSD mice, following challenge with the HexM protein. However, it was not the case for the HexM-specific T cell response. This might be explained by a ‘linked recognition’ mechanism of immune stimulation, in which the activation of T cell-dependent antibodies is initiated by helper T cells [43]. In this case, a higher T cell response would be expected earlier in the infection stage. This is because previously naïve B cells would be activated by accessory signals from armed helper T cells that recognize the antigen, thus leading to the activation and release of antibodies.

To further study the specificity of the T cell response in the TSD mice, the ELISpot assay was repeated using cryo-preserved splenocytes. Repeating the assay provided an opportunity to explore the nature of the immune response, by separating the total HexM pool into the three separate pools. In addition to the T-cell response against novel HexM epitopes (i.e., not homologous with either mouse or human HexA), it was also noted that a smaller, but statistically significant, T-cell response to the HexM peptide pool with 100% homology between human and mouse (Figure 1C), was present as well. Surprisingly, this was the case even in Het mice that express endogenous mouse HexA. Our study did not formally delineate the relative contributions of the HexM- and HEXA-specific epitopes to the overall immune response. Moreover, it is not clear why the HexA epitopes that are conserved between mice and humans still stimulated T cells in Het mice. However, it is postulated that the immune response to neighboring HexM epitopes, or the AAV9 vector, may act as an adjuvant for immune stimulation. Vehicle-injected mice did not show a T-cell response against these mouse-human conserved HexA epitopes. Therefore, we can speculate that the immune response against the overall HexM protein stimulated this “autoimmune” response against these conserved HexA epitopes.

Although the AAV vector has a relatively low pro-inflammatory profile [44], previous studies have shown a negative effect of pre-existing immunity to AAV on the efficiency of the gene therapy treatment [39,45,46]. Even low levels of neutralizing antibodies to AAV can counteract large doses of the vector [25,37]. In this study, the data obtained from AAV9 capsid ELISpots and neutralizing antibody assays revealed that the mice did mount an immune response to the AAV9 capsid. There was a high T cell response to the AAV9 capsid observed in TSD mice after gene therapy treatment compared to vehicle injected mice. Unfortunately, this assay was not repeated using splenocytes isolated from SD mice, due to a shortage of cells. There was also the presence of high levels of neutralizing antibodies to the AAV9 capsid in the sera of TSD and SD mice given scAAV9-*HEXM*, compared to the vehicle (Supplementary Tables S2 and S3). The intravenous administration of the AAV vector does have a high likelihood of provoking an immune response, therefore these results were expected.

The biodistribution of HexM DNA revealed that the gene therapy reached the liver and brain in both mouse models, and that the immune response to the treatment was accompanied by a significant decrease in genome persistence in the liver of treated TSD mice. This could be the result of the immune cells attacking the human derived transgene product as well as the viral capsid. Consequently, the transduced cell population would diminish, thereby decreasing the efficiency of the treatment. A low biodistribution due to an immune response to gene therapy was also evident in a study assessing the immune response following AAV9-GFP injections in non-human primates (NHPs) [42]. It was observed that GFP-treated NHPs demonstrated a decrease in GFP expression in the lumbar spinal cord over time. GFP expression was sustained only when animals were treated with an immunosuppressant, to diminish the anti-GFP immune response. Although the correlation between antibody levels in the sera and vector genomes in the liver and in the brain were not statistically significant, the observations obtained from our study are a clear indication that the immune response to HexM and the AAV9 capsid could contribute to either the failure or decreased long-term benefit of the scAAV9-*HEXM* gene therapy treatment. In our study, it should be noted that the decrease in the liver distribution could also be due to other toxicity mechanisms related to the high level of liver transduction.

We acknowledge several limitations of our study. The study design was slightly different in TSD and SD mice. However, even though the TSD and SD studies were done independently in separate labs, both showed similar results. We also acknowledge that we did not assess the long-term immune response to HexM, to observe if there was any tolerization that could have been generated, even after the initial immune response. However, the objective of this study was to assess the immunocompetence of TSD and SD mouse models in the investigation of immune responses to the *HEXM* gene transfer. In future experiments, detailed T-cell subset analysis to examine specific immune cells is warranted. Another small limitation is that we studied only the intravenous route of vector administration and no other route of delivery. The rationale was to create a normal immune response of the body. Delivery of the vector directly into slightly immune-privileged compartments like brain parenchyma or into cerebrospinal fluid (via either lumbar puncture or intracerebroventricular or cisterna magna injection) can have advantages as an alternative to the IV route and shall be studied in future optimization studies but were not considered best for this immunocompetency study.

The presence of an immune response to the therapeutic protein HexM and to the AAV9 viral capsid could have detrimental effects on scAAV9-*HEXM* gene delivery as a treatment for GM2 gangliosidoses. Further, it should be noted that this type of immune response could pertain not only to HexM, but also other attempts to deliver HexA or HexB into patients who lack the respective native enzyme, such as in the infantile forms of the diseases. It is imperative to formulate strategies to either prevent this unwanted immune response, or to tolerize the patient to the therapeutic protein and viral capsid. A method for inducing immune tolerance in a patient would allow a lower dose of the treatment to be used while producing a persistent therapeutic result. Using a lower dose would also decrease the potential for toxicity and reduce the cost of the treatment. Therefore, the use of immunomodulatory gene therapy may be essential for the long-term treatment of GM2 gangliosidosis disorders.

## 4. Materials and Methods

### 4.1. Experimental Animal Models

The TSD and SD mouse models have been previously described [6]. Both models were obtained from The Jackson Laboratory.

For the TSD mouse model, heterozygous (Het, *Hexa*^(+/−)^) and homozygous knockout (KO, *Hexa*^(−/−)^) TSD mice were used in this study [47]. Ear clippings from the mice were tested using PCR to determine the *Hexa* genotype. Study mice were euthanized at 11 weeks of age (*n* = 24) or 14 weeks of age (*n* = 24). All animal care and procedures for TSD mice were in compliance with the Guide for the Care and Use of Laboratory animals, and approved by the University of North Carolina Institutional Animal Care and Usage Committee.

For the SD mouse model, Het (*Hexb*^(+/−)^) and KO (*Hexb*^(−/−)^) mice were used, and the *Hexb* genotype was also determined using PCR genotypic analysis on ear clippings, obtained from the mice. Experimental mice were euthanized at 9 weeks (*n* = 3) or 15 weeks (*n* = 34) of age. All animal care and procedures for SD mice were in compliance with the Canadian Council on Animal Care regulations and received prior approval by the University Animal Care Committee at Queen’s University, Kingston, ON, Canada.

### 4.2. Construction of scAAV9-HEXM

The construction of the scAAV9-*HEXM* vector was as previously described [12,30]. The *HEXM* transgene was inserted into the scAAV backbone previously described [31] and includes a short sequence length synthetic ubiquitous UsP promoter [12,48]. The viral vectors were generated at University of North Carolina, NC, USA.

### 4.3. Study Design

**Figure 8:**
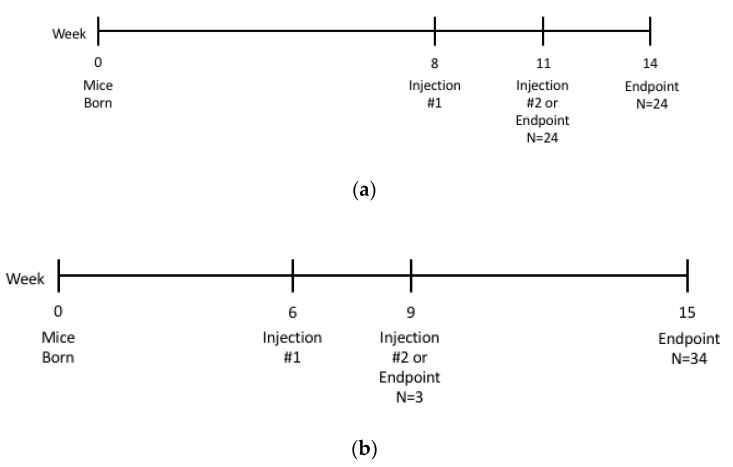
Study timeline in weeks of (a) TSD study and (b) SD study. (**a**) TSD mice were given the first injection at 8 weeks of age, and the second injection at 11 weeks of age. Mice were euthanized either at 11 weeks (*n* = 24) or at 14 weeks (*n* = 24). (**b**) SD mice were given their first injection at 6 weeks of age and their second injection at 9 weeks of age. Mice were euthanized at 9 weeks (*n* = 3) or at 15 weeks (*n* = 34).

Figure 8 shows the study timeline, including birth, age of injections, and endpoints for both TSD (8A) and SD (8B) mice. All manipulations of TSD mice were conducted at the University of North Carolina, and all manipulations of SD mice were conducted at Queen’s University.

Table 1 shows the study design for TSD mice. TSD mice (cohorts 5–8), including KO mice (*n* = 12) and Het mice (*n* = 12), received an intravenous scAAV9-*HEXM* vector injection at eight weeks of age. Out of this, six Het mice and six KO mice (cohorts 5 and 6) were euthanized three weeks post injection #1. The remaining six Het and six KO mice received an injection of HexM-His_6_ purified protein (50 μg per mouse), mixed with complete Freund’s adjuvant (CFA), through subcutaneous administration at ~11–12 weeks of age. These mice were then euthanized three weeks post injection #2. Control cohorts (1–4), with Het (*n* = 12) and KO (*n* = 12) mice, were injected intravenously with vehicle alone (Phosphate Buffered Saline, PBS, with 350 mM total NaCl and 5% sorbitol) at 8 weeks of age. Six Het mice and six KO mice were euthanized three weeks post injection, and the remaining mice were euthanized six weeks post injection.

Table 2 shows the study design of SD mice. Cohorts 1–6 included negative and positive control SD mice, KO and Het. Cohorts 1 and 2, Het (*n* = 3) and KO (*n* = 3), respectively, received an equivalent volume of vehicle (PBS with 350 nM total NaCl and 5% sorbitol) to act as a negative control, with CFA in the first injection and Incomplete Freund’s Adjuvant (IFA) in second injection. Cohort 3, KO (*n* = 3), received vehicle alone with no adjuvant at all. Mice in Cohort 4, Het (*n* = 4), and cohort 5, KO (*n* = 6), were injected with purified HexM-His_6_ protein and CFA at 6 weeks, followed by an injection of purified HexM-His_6_ with IFA at 9 weeks of age. Cohort 6, KO *n* = 6, received the same treatment as cohorts 4 and 5, but without adjuvant. Cohorts 7–10 included SD KO mice (*n* = 12) that received 1 × 10^12^ vg of the scAAV9-*HEXM* vector injected intravenously, through the tail vein, at 6 weeks of age. Of these 12 mice, 6 received no second injection, with 3 out of those 6 (cohort 7) euthanized three weeks post injection, and the remaining 3 (cohort 8) euthanized nine weeks later. Of the remaining six KO mice injected with scAAV9-*HEXM*, three mice (cohort 9) received a booster intravenous injection of scAAV9-*HEXM*, and three mice (cohort 10) received an intraperitoneal injection of pure HexM-His_6_ protein, without any adjuvant, at nine weeks of age. These mice were euthanized at 15 weeks of age. All control mice were euthanized at 15 weeks of age.

### 4.4. Tissue and Serum Processing

Blood and sera from TSD and SD (KO and Het) mice were obtained pre-treatment and at the termination time points. For pre-treatment blood collection time points, the blood was obtained from the saphenous vein. Blood collection at the endpoints was done by cardiac puncture. Blood was then spun down at 3500 revolutions per minute (rpm) for 10 min in a low-speed centrifuge. Serum was separated from the remaining blood components. Tissues for immunological, biochemical, and molecular analyses were collected at termination. These included brain, liver, spleen, inguinal, and mesenteric lymph nodes. Splenocytes and lymph node cells were isolated from the spleens at the termination time point and used fresh for cytokine analysis (for TSD mice), or cryo-preserved for further analysis.

### 4.5. Indirect Enzyme-Linked Immunosorbent Assay

The indirect enzyme-linked immunosorbent assay (ELISA) technique was used to quantify the presence of antibodies to the HexM therapeutic protein in the sera of experimental mice. A standard curve was developed using 5 μg/mL of HexM-His_6_ protein (purified separately by the Walia lab) as the antigen, known concentrations of sheep anti-HEXA (obtained from Dr. Don Mahuran, SickKids Hospital, Toronto, ON, Canada) as the primary antibody, and donkey anti-sheep conjugated antibody (1:5000, AB324P; Chemicon International, Temecula, CA, USA) as the secondary antibody. To test the presence of antibodies in the mice, the sera was used as primary antibody and donkey anti-mouse conjugated antibody (1:5000, AP192P, Chemicon International) was used as the secondary antibody. Turbo TMB-ELISA (cat. No. 34022, Pierce, Rochford, IL, USA) substrate solution was then added to the wells for development, followed by a stop solution (0.2 M sulphuric acid). Plates were read at 450 nm absorbance using a Thermo Electron Corporation Varioskan plate reader.

### 4.6. IFN-γ Analysis: Enzyme-Linked Immunospot Assay

Interferon-γ Enzyme-Linked Immunospot (ELISpot) assays were carried out using freshly prepared or cryo-preserved single cell suspensions of splenocytes and pooled lymphocytes (inguinal and mesenteric). This assay was developed using a PEPscreen^®^ peptide library covering the entire HexM amino acid sequence, including fifty-two peptides overlapped by 10 amino acids (Supplementary Table S1). This peptide pool library was used to stimulate CD4^+^ and CD8^+^ T cells in the spleen to secrete the IFN-γ cytokine.

The splenocytes obtained from TSD mice were tested in three separate pools (Table 3) derived from the fifty-two HexM peptide library, as well as all the peptides in the HexM library. The first pool consisted of the 15 HexM-specific peptides that are specific to the structural modifications designed to make HexM and therefore are not present in human HexA. The second pool consisted of five mouse-human 100% homology peptides that are completely conserved between mouse and human HexA. The third pool consisted of the remaining 32 peptides that are specific to human HexA. Splenocytes obtained from SD mice were tested using the total pool of the fifty-two HexM peptides.

MultiScreen IP Filter plates (MSIPS4510, Millipore, Bedford, MA, USA) and mouse IFN-γ ELISpot kits (cat. No. 88-7384, eBioscience, San Diego, CA, USA) were used in this protocol. The positive control wells were stimulated by a cell stimulation cocktail containing PMA and ionomycin (cat. No. 00-4970-03, eBioscience, San Diego, CA, USA). The negative control wells included 0.32% dimelthyl sulfoxide (DMSO), the solvent used to resuspend the peptides in the library. No-cell wells were also included to determine the background caused by the reagents used in the assay. The cells were cultured for 48 h in the presence of the stimuli in a filter plate previously coated with the IFN-γ capture antibody. After 48 h, the plates were processed for the detection of spot development. The ‘number of spots’ in a well is a measure of the number of T-cells secreting IFN-γ in response to the specific stimulus provided. Plates were evaluated by ZellNet Consulting, Inc. (Fort Lee, NJ, USA) with a KS Elispot Reader System (Carl Zeiss, Inc., Thornwood, NY, USA) using KS Elispot software version 4.9.16. Reading parameters were established following international harmonization guidelines [49].

This assay was repeated using the AAV9 capsid PEPscreen^®^ peptide library to assess the T cell response to the AAV9 capsid in TSD mice. Because this entire AAV9 capsid library is novel relative to the mouse genome, only the total peptide pool was tested. The assay was done on fresh splenocytes from TSD mice and on once frozen-thawed splenocytes from SD mice.

### 4.7. Quantitative PCR Analysis of Vector Genomes

Analysis of vector biodistribution was done by quantitative PCR (qPCR). Tissue DNA was purified and quantified by adopting a previously described method [23] but using primers for *HEXM.* Data are reported as the number of double-stranded *HEXM* sequences per 2 double-stranded copies of the murine LaminB2 locus, or in other words, the number of vector DNA copies per diploid mouse genome.

### 4.8. AAV9 Capsid Neutralizing Antibodies Assay

A cell-based assay was done to determine the titer of AAV9 capsid neutralizing antibodies in the sera of mice from this study. Briefly, Lec2 cells (Catalog #CRL-2972; ATCC, Manassa, VA, USA) were cultured in 48-well plates and infected with scAAV9-CBh-GFP virus that had been incubated with different dilutions of sera (two times serial dilutions from 1:500 to 1:32,000) from mice treated in the study. After 48 h of culture, the GFP expression levels in each well was assessed in comparison to the positive control well (virus only, no serum incubation) to determine the titer. The neutralization antibody titer is the dilution at which there is a 50% reduction in transduction when compared to the positive control. The cells were collected and analyzed by flow cytometry to quantitatively determine the titer.

### 4.9. Statistical Analysis

Multi-factorial ANOVAs (Male versus Female, Het versus KO, Vehicle versus scAAV9-HexM, and 3- versus 6-week harvest of splenocytes) were performed on the ELISpot results from the TSD mice, after logarithmic transform of the spot counts to reduce the non-normality of the distributions (analysis software NCSS 12 version 12.0.12, general linear model for fixed factors). Two-way ANOVAs with Tukey’s multiple comparison tests were performed on the results obtained from the ELISA, and qPCR assays in TSD mice. One-way ANOVAs with Tukey’s multiple comparison tests were performed on the log-transformed ELISpot data, and on the ELISA and qPCR results of the SD mice.

## Figures and Tables

**Table 1:** Study design for TSD mice, including genotype, number of mice, injection, and termination time points.

Cohort	Genotype	*n* =	Step 1 (Age ~8 Weeks)			Step 2 (Age ~11 Weeks)			Termination Age
			Injection 1	Dose	Adjuvant	Injection 2	Dose	Adjuvant	
1	Het	3M/3F	Vehicle	-	None	-	-	-	11 Weeks (3 weeks post injection 1)
2	KO	3M/3F	Vehicle	-	None	-	-	-	11 Weeks (3 weeks post injection 1)
3	Het	3M/3F	Vehicle	-	None	-	-	-	14 Weeks (6 weeks post injection 1)
4	KO	3M/3F	Vehicle	-	None	-	-	-	14 Weeks (6 weeks post injection 1)
5	Het	3M/3F	scAAV9-*HEXM*	1.0 × 10^12^ vg	None	-	-	-	11 Weeks (3 weeks post injection 1)
6	KO	3M/3F	scAAV9-*HEXM*	1.0 × 10^12^ vg	None	-	-	-	11 Weeks (3 weeks post injection 1)
7	Het	3M/3F	scAAV9-*HEXM*	1.0 × 10^12^ vg	None	HexM Protein	50 μg	CFA	14 Weeks (6 weeks post injection 1)
8	KO	3M/3F	scAAV9-*HEXM*	1.0 × 10^12^ vg	None	HexM Protein	50 μg	CFA	14 Weeks (6 weeks post injection 1)

**Table 2:** Study design for SD mice, including genotype, number of mice, injections, and termination time points.

Cohort	Genotype	*n* =	Step 1 (Age ~8 Weeks)			Step 2 (Age ~11 Weeks)			Termination Age
			Injection 1	Dose	Adjuvant	Injection 2	Dose	Adjuvant	
1	Het	3	Vehicle	-	CFA	Vehicle	0	IFA	15 Weeks (6 weeks post injection 2)
2	KO	3	Vehicle	-	CFA	Vehicle	0	IFA	15 Weeks (6 weeks post injection 2)
3	KO	3	Vehicle	-	None	Vehicle	0	None	15 Weeks (6 weeks post injection 2)
4	Het	4	HexM Protein	25 μg	CFA	HexM Protein	25 μg	IFA	15 Weeks (6 weeks post injection 2)
5	KO	6	HexM Protein	25 μg	CFA	HexM Protein	25 μg	IFA	15 Weeks (6 weeks post injection 2)
6	KO	6	HexM Protein	25 μg	None	HexM Protein	25 μg	None	15 Weeks (6 weeks post injection 2)
7	KO	3	scAAV9-*HEXM*	1 × 10^12^ vg	None	-	-	-	9 Weeks (3 weeks post injection 1)
8	KO	3	scAAV9-*HEXM*	1 × 10^12^ vg	None	-	-	-	15 Weeks (9 weeks post injection 1)
9	KO	3	scAAV9-*HEXM*	1 × 10^12^ vg	None	scAAV9-*HEXM*	1 × 10^12^ vg	None	15 Weeks (9 weeks post injection 1)
10	KO	3	scAAV9-*HEXM*	1 × 10^12^ vg	None	HexM Protein	25 μg	None	15 Weeks (9 weeks post injection 1)

**Table 3:** Composition of HexM Peptide Pools.

	# Peptides in the Pool	100% Homology to Human HexA	100% Homology to Mouse HexA
Novel HexM Peptides	15	No	No
Mouse-Human Homologous HexA Peptides	5	Yes	Yes
Human-Specific HexA Peptides	32	Yes	No

## Data Availability

Data can be obtained on request from the PI.

## Supplementary Materials

The following are available online at https://www.mdpi.com/article/10.3390/ijms22136751/s1, Figure S1: CFSE dilution assay to detect proliferating CD8^+^ T cells in response to HexM-specific stimuli. Figure S2: Neutralization antibodies against AAV9 capsid in TSD mice sera at 3 weeks and 6 weeks post scAAV9-HEXM injection. Table S1: Prospector PEPscreen^®^ Custom Peptide Library for HexM Isoenzyme. Table S2: AAV9 capsid neutralizing antibody titers in TSD mice sera at 3 weeks and 6 weeks post scAAV9-*HEXM* injection. Table S3: AAV9 capsid neutralizing antibody titers in SD mice sera at 3 weeks and 10 weeks post scAAV9-*HEXM* injection.
