# Supplementary material for: Investigating Immune Responses to the scAAV9-HEXM Gene Therapy Treatment in Tay–Sachs Disease and Sandhoff Disease Mouse Models
Source: Int J Mol Sci. 2021 Jun 23;22(13):6751. doi: 10.3390/ijms22136751 (PMC8268035; doi:10.3390/ijms22136751)
Supplement: Supplementary file 1 [file ijms-22-06751-s001.zip › Immunology Paper I Supplementary Materials.pdf]

SUPPLEMENTARY MATERIAL

Proliferative response to HexM and AAV9 capsid in TSD mice

Carboxyfluorescein succinimidyl ester (CFSE) Dilution Assay

A CFSE dilution assay was used to evaluate the proliferative response of $CD8^+$ T cells from the TSD mice in the study. Briefly, splenocytes were stained with CFSE, a nuclear dye, and then cultured in the presence of the HexM or AAV9 capsid peptide pools for 5 days. On the fifth day, they were collected, stained for CD8, fixed, and analyzed by flow cytometry on the CyAn ADP flow cytometer (Dako Cytomation, Glostrup, Denmark). With each division, CFSE divides evenly between the two daughter cells, therefore proliferation can be quantified by observing cells with reduced CFSE intensity.

According to the data obtained from the CFSE dilution assay, the $CD8^+$ T cell proliferation in response to stimulation with HexM peptide pools was very low or absent in samples taken 3 weeks post injection. However, in some groups of mice, there was an increase in the proliferative response in samples taken 6 weeks post injection (Supplementary Figure S1).

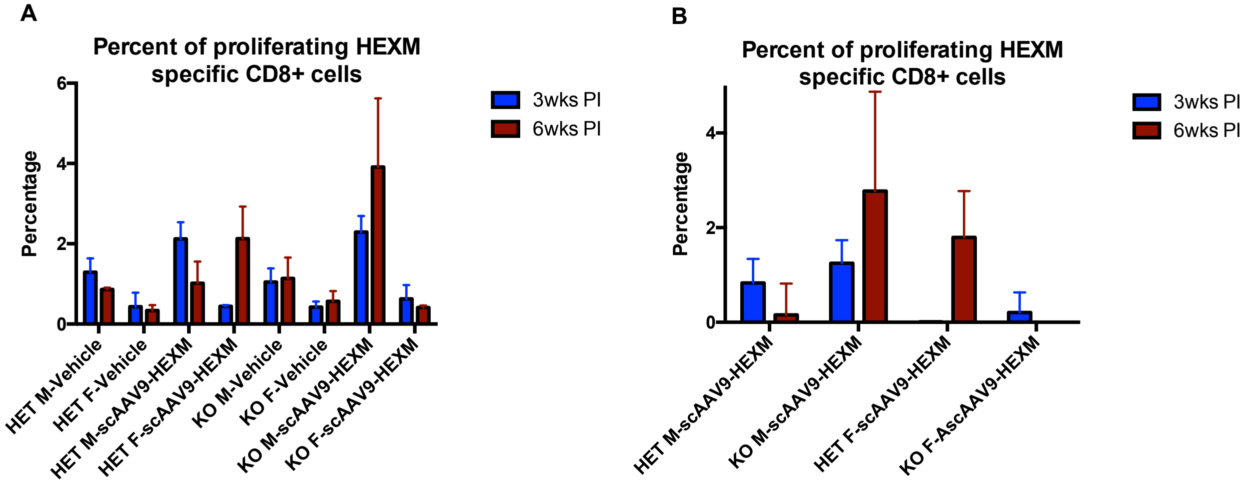

Supplementary Figure S1. CFSE dilution assay to detect proliferating $CD8^+$ T cells in response to HexM-specific stimuli.

Percent of proliferating HexM-specific $CD8^+$ T cells. (A) Comparison of scAAV9-*HEXM* treated to vehicle injected mice. (B) Comparison of KO and Het mice. $CD8^+$ T cell proliferation against HexM was very low in samples taken 3 weeks post injection (PI). A larger proliferative response was observed in samples taken at the later time point (6 weeks PI) in some mice. M= male, F= female, wks= weeks, PI= post injection.

Neutralizing antibodies against AAV9 capsid in the sera of both <u>TSD and SD</u> mice

In contrast to vehicle injected mice, which had no AAV9 capsid neutralizing antibodies, all the scAAV9-*HEXM*-injected mice had neutralizing antibodies against the capsid at similar levels (Supplementary Figure S2, Supplementary Tables S2 and S3). These results are consistent with the expectation that mice will develop anti-AAV9 antibodies following intravenous administration of an AAV9 vector.

<u>3 wks PI of scAAV9-*hHEXM*</u>

HET

M-4-1:4000

M-5-1:32000

M-6-1:2000

KO

M-10-1:2000

M-11- 1:2000

M-12-1:8000

<u>6 wks PI of scAAV9-*hHEXM*</u>

HET-Vehicle

M-25-<1:500

M-26- <1:500

M-27- <1:500

HET-scAAV9/*hHEXM*

M-28-1:1000

M-29-1:4000

M-30-1:2000

KO-Vehicle

M-31- <1:500

M-32- <1:500

M-33- <1:500

KO-scAAV9/*hHEXM*

M-34- 1:1000

M-35- 1:1000

M-36-1:2000

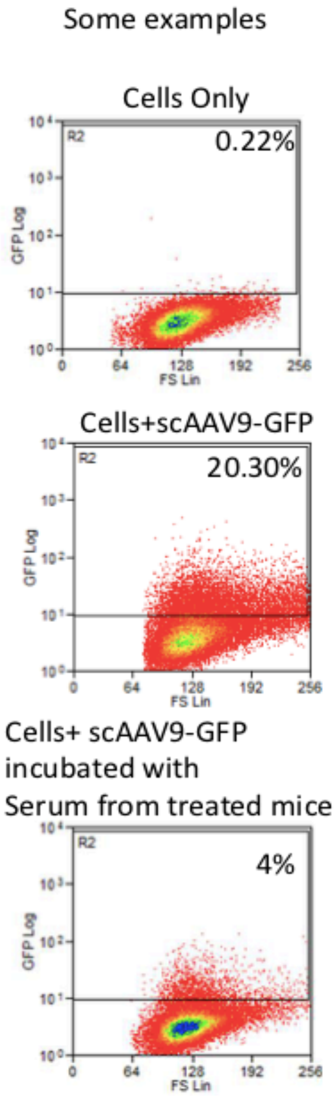

Supplementary Figure S2. Neutralization antibodies against AAV9 capsid in TSD mice sera at 3 weeks and 6 weeks post scAAV9-*HEXM* injection. M-nn = mouse ID number, M= male, wks= weeks, PI= post injection. The examples show the flow cytometry results for GFP expression in LEC2 cells cultured without any AAV treatment (top panel), for cells treated with scAAV9-CBh-GFP (middle panel), and the reduced percentage of GFP-expressing LEC2 cells when treated with scAAV9-GBh-GFP in the presence of serum from treated mice (bottom panel), indicating reduced cell transduction due to AAV9 neutralizing antibodies in the mouse serum.

Weeks Post-Injection	Mouse ID	Genotype	Injection	Neutralizing Ab Titer
3	M-4	Het	scAAV9-*HEXM*	1:4000
	M-5			1:32000
	M-6			1:2000
	M-10	KO		1:2000
	M-11			1:2000
	M-12			1:8000
6	M-25	Het	Vehicle	<1:500
	M-26			<1:500
	M-27			<1:500
	M-28	Het	scAAV9-*HEXM*	1:1000
	M-29			1:4000
	M-30			1:2000
	M-31	KO	Vehicle	<1:500
	M-32			<1:500
	M-33			<1:500
	M-34	KO	scAAV9-*HEXM*	1:1000
	M-35			1:1000
	M-36			1:2000

Supplementary Table S2. AAV9 capsid neutralizing antibody titers in TSD mice sera at 3 weeks and 6 weeks post scAAV9-*HEXM* injection. M-nn = mouse ID number, M= male, neutralizing Ab titer = the dilution of mouse serum at which there was a 50% reduction in transduction of LEC2 cells compared to the LEC2 cells treated with scAAV9-CBh-GFP alone.

Weeks Post-Injection	Mouse ID	Genotype	Injection 1	Injection 2	Neutralizing Ab Titer
3	764	KO	scAAV9-HEXM	-	1:500
	762				1:1000
	758				1:1000
10	632	KO	PBS	PBS	<1:500
	635				<1:500
	624				<1:500
	710		scAAV9-HEXM	HexM	1:2000
	711				1:1000
	712		scAAV9-HEXM	scAAV9-HEXM	1:1000
	716				1:2000
	765				1:500
	717		scAAV9-HEXM	-	1:2000
	719				1:2000
	720		HexM+Adjuvant	HexM+Adjuvant	<1:500
	731				<1:500
	733				<1:500
	736		HexM	HexM	<1:500
	737				<1:500
	739				<1:500
	747	Het	PBS+Adjuvant	PBS+Adjuvant	<1:500
	772				<1:500
	774				<1:500
	763		HexM+Adjuvant	HexM+Adjuvant	<1:500
	761				<1:500
	759				<1:500

Supplementary Table S3. AAV9 capsid neutralizing antibody titers in SD mice sera at 3 weeks and 10 weeks post scAAV9-*HEXM* injection. M-nn = mouse ID number, M= male, neutralizing Ab titer = the dilution of mouse serum at which there was a 50% reduction in transduction of LEC2 cells compared to the LEC2 cells treated with scAAV9-CBh-GFP alone.
