# Supplementary material for: Investigating Immune Responses to the scAAV9-HEXM Gene Therapy Treatment in Tay–Sachs Disease and Sandhoff Disease Mouse Models
Source: Int J Mol Sci. 2021 Jun 23;22(13):6751. doi: 10.3390/ijms22136751 (PMC8268035; doi:10.3390/ijms22136751)
Supplement: Supplementary file 1 [file ijms-22-06751-s001.zip › Supplementary Table S1.pdf]

Peptide ID	Position	Sequence
1	1-22	MTSSRLWFSLLLAAAFAGRATA
2	11-30	LLAAAFAGRATALWPWPQNF
3	23-42	LWPWPQNFQTSDQRYVLYPN
4	33-52	SDQRYVLYPNNFQFQYDVSS
5	43-62	NFQFQYDVSSAAQPGCSVLD
6	53-74	AAQPGCSVLDEAFQRYRDLLFG
7	68-83	EAFQRYRDLLFGSGSWPRPYL
8	75-88	SGSWPRPYLTGKRH
9	80-99	RPYLTGKRHTLEKNVLVVSV
10	89-108	TLEKNVLVVSVVTPGCNQLP
11	99-118	VVTPGCNQLPTLESVENYTL
12	109-128	TLESVENYTLTINDDQCLLL
13	119-138	TINDDQCLLLSETVWGALRG
14	129-148	SETVWGALRGLETFSQLVWK
15	139-158	LETFSQLVWKSAEGTFFINK
16	149-168	SAEGTFFINKTEIEDFPRFP
17	159-178	TEIEDFPRFPHRGLLLDTSR
18	169-188	HRGLLLDTSRHYLPLKSILD
19	179-198	HYLPLKSILDTLDVMAYNKL
20	189-208	TLDVMAYNKLNVFHWHLVDD
21	199-218	NVFHWHLVDDQSFPYESFTF
22	209-228	QSFPYESFTFPELMRKGSYS
23	219-238	PELMRKGSYSLSHIYTAQDV
24	229-248	LSHIYTAQDVKEVIEYARLR
25	239-258	KEVIEYARLRGIRVLAEFDT
26	249-268	GIRVLAEFDTPGHTLSWGPG
27	259-278	PGHTLSWGPGIPGLLTPCYS
28	269-288	IPGLLTPCYSGSEPSGTFGP
29	279-298	GSEPSGTFGPVNPSLNNTYE
30	289-308	VNPSLNNTYEFMSTFFLEVS
31	299-318	FMSTFFLEVSSVFPDFYLHL
32	309-328	SVFPDFYLHLGGDEVDFTCW
33	319-338	GGDEVDFTCWKSNPEIQDFM
34	329-348	KSNPEIQDFMRKKGFGEDFK
35	339-358	RKKGFGEDFKQLESFYIQTL
36	349-368	QLESFYIQTLLDIVSSYGKG
37	359-378	LDIVSSYGKGYVVWQEVFDN
38	369-388	YVVWQEVFDNKVKIQPDTII
39	379-398	KVKIQPDTIIQVWREDIPVN
40	389-408	QVWREDIPVNYMKELELVTK
41	399-418	YMKELELVTKAGFRALLSAP
42	409-428	AGFRALLSAPWYLNRISYGQ
43	419-438	WYLNRISYGQDWRKFYKVEP

44	429-448	DWRKFYKVEPLAFEGTPEQK
45	439-458	LAFEGTPEQKALVIGGEACM
46	449-468	ALVIGGEACMWGEYVDATNL
47	459-478	WGEYVDATNLVPRLWPRAGA
48	469-488	VPRLWPRAGAVAERLWSNKL
49	479-498	VAERLWSNKLTRDMDDAYDR
50	489-508	TRDMDDAYDRLSHFRCELVR
51	499-518	LSHFRCELVRRGVAAQPLYA
52	509-528	RGVAAQPLYAGYCNQEFEQT

Supplementary Table S1. Prospector PEPscreen® Custom Peptide Library for HexM Isoenzyme.
Blue- Peptide Pool #1 (Peptides unique to µ portion of HexM)
Green- Peptide Pool #2 (Peptides 100% homologous between human and mouse HEXA
Orange- Peptide Pool #3 (Peptides with some homology between human and mouse HEXA)
